# Intracranial pressure monitoring in posterior fossa lesions—systematic review and meta-analysis

**DOI:** 10.1007/s10143-022-01746-y

**Published:** 2022-02-03

**Authors:** Sae-Yeon Won, Daniel Dubinski, Jonas Hagemeier, Bedjan Behmanesh, Svorad Trnovec, Joshua D. Bernstock, Thomas M. Freiman, Florian Gessler

**Affiliations:** 1 Department of Neurosurgery, University Hospital Rostock, Schillingallee 35, 18057 Rostock, Germany; 2 Department of Neurosurgery, Brigham and Women’s Hospital, Harvard Medical School, Boston, USA

**Keywords:** Intracranial pressure monitoring, Posterior fossa lesion, Supratentorial ICP measurement, Infratentorial ICP measurement, Transtentorial gradient

## Abstract

Elevated intracranial pressure (ICP) with reduced cerebral perfusion pressure is a well-known cause of secondary brain injury. Previously, there have been some reports describing different supra- and infratentorial ICP measurements depending on the location of the mass effect. Therefore, we aimed to perform a systematic review and meta-analysis to clarify the issue of optimal ICP monitoring in the infratentorial mass lesion. A literature search of electronic databases (PUBMED, EMBASE) was performed from January 1969 until February 2021 according to the Preferred Reporting Items for Systematic Reviews and Meta-analysis (PRISMA) statement. Two assessors are independently screened for eligible studies reporting the use of simultaneous ICP monitoring in the supra- and infratentorial compartments. For quality assessment of those studies, the New Castle Ottawa Scale was used. The primary outcome was to evaluate the value of supra- and infratentorial ICP measurement, and the secondary outcome was to determine the time threshold until equalization of both values. Current evidence surrounding infratentorial ICP measurement was found to be low to very low quality according to New Castle Ottawa Scale. Eight studies were included in the systematic review, four of them containing human subjects encompassing 27 patients with infratentorial pathology. The pooled data demonstrated significantly higher infratentorial ICP values than supratentorial ICP values 12 h after onset (*p* < 0.05, 95% CI 3.82–5.38) up to 24 h after onset (*p* < 0.05; CI 1.14–3.98). After 48–72 h, both ICP measurements equilibrated showing no significant difference. Further, four studies containing 26 pigs and eight dogs showed a simultaneous increase of supra- and infratentorial ICP value according to the increase of supratentorial mass volume; however, there was a significant difference towards lower ICP in the infratentorial compartment compared to the supratentorial compartment. The transtentorial gradient leads to a significant discrepancy between supra- and infratentorial ICP monitoring. Therefore, infratentorial ICP monitoring is warranted in case of posterior fossa lesions for at least 48 h.

## Introduction

Increased intracranial pressure (ICP) with reduced cerebral perfusion pressure are long-established and well-known causes of secondary brain injury associated with poor clinical outcome [1]. Among a variety of ICP measuring devices, the most commonly used include ventricular catheter or intraparenchymal ICP probe. Irrespective of the location of the pathology or mass effect, it is a clinical practice that a single location for ICP measurement reflects an accurate pressure throughout the brain [2]. In view of the anatomic subdivision of our intracranial compartments by the falx or tentorium, the question arises if the ICP value is reliably independent from the location of the mass and ICP monitoring. Interestingly, the standardized clinical management of infratentorial mass lesions (cerebellar hemorrhage, stroke, tumor) relies on the supratentorial ventricular ICP measurement [3–5]. Previously, there have been several reports of patients, cadavers, and primates showing significant differences of ICP values depending on the location of mass and ICP monitoring. We therefore aimed to perform a systematic review and meta-analysis of the literature focusing on supra- and infratentorial ICP measurements to clarify the issue of intracranial compartments and ICP monitoring in case of infratentorial mass lesions [6–14].

## Methods

### Search strategy

The meta-analysis was conducted in accordance with the Preferred Reporting items for Systematic Reviews and Meta-analysis (PRISMA) statement. We systematically searched the PubMed, Embase, Web of Science, and Cochrane databases including manuscripts published between January 1969 and February 2021, with language restricted to English, and identified all studies related to the use of simultaneously ICP monitoring in the supra- and infratentorial compartment. The literature was searched by using the predefined keywords “posterior fossa AND Intracerebral pressure monitoring,” “infratentorial pressure monitoring,” “posterior fossa pressure monitoring,” “cerebellum AND intracranial pressure monitoring,” and “cerebellar pressure monitoring.”

### Data extraction and quality assessment

**Fig. 1:**
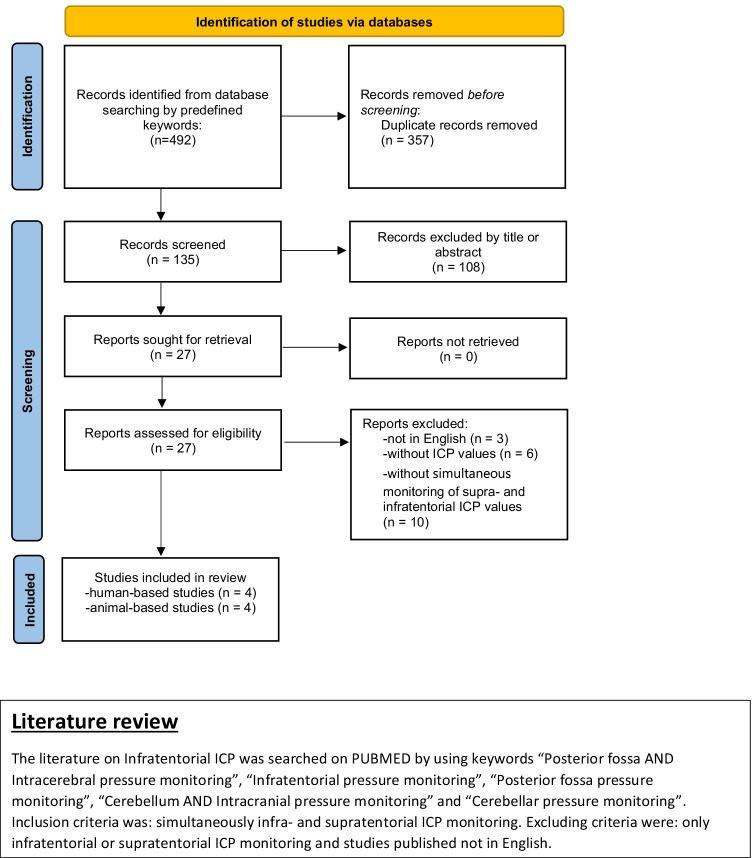
Flowchart outlining of systematic review selection criteria

Two assessors (S.W. and J.H.) independently screened for eligible studies by title and abstract. After prescreening, both assessors reviewed the full manuscripts of 27 eligible studies. Three studies in a language other than English, six studies without reporting of ICP values, and ten studies without simultaneous monitoring of supra- and infratentorial pressure monitoring were excluded. In total, eight studies were included in the systematic review, containing four studies with humans and four studies with primates (Fig. 1) [2, 7–12, 14]. Data were extracted in standardized data collection forms. The extracted information included the following items: first author name, year of publication, impact factor of the journal, sample size, species, pathology/illness, simultaneously supra- and infratentorial measured ICP values, and the duration of ICP monitoring. For the quality assessment of those studies, the New Castle Ottawa Scale was used by two independent reviewers.

### Outcome measures

The primary outcome was to evaluate the value of supratentorial and infratentorial ICP measurement in different neurosurgical conditions. The secondary outcome was to determine the time threshold until equalization of both supra- and infratentorial ICP values. Therefore, we categorized the data into 3 time periods depending on the duration of ICP measurement (0–12 h, 0–24 h, > 48–72 h) and compared both ICP measurements.

### Statistical analysis

Review Manager 5.4.1 (Cochrane Collaboration, Copenhagen, Denmark) was used to perform the meta-analysis. Supra- and infratentorial ICP values were summarized for the included studies using pooled weighted means with standard deviation (SD). The I^2^ statistic was used to reveal the heterogeneity of treatment effects. I^2^ of 0% indicates no heterogeneity, 25% indicates slight heterogeneity, 50% indicates middle heterogeneity, and 75% indicates high heterogeneity [15]. All statistical analysis were two-sided, and a *p* value of < 0.05 was considered as statistically significant.

## Results

### Study characteristics

Eight of 26 studies were included in the systematic review, four of them contained human subjects [7, 8, 11, 12]. Two studies were case series and the other two were case reports, which were included in the meta-analysis resulting in a total of 27 patients. All patients had an intracranial pathology in the infratentorial compartment as described in Table 1.

**Table 1:** Study characteristics (humans)

References	Year	Design	Patients (*n*)	Diagnosis	Quality (NOS)	Impact factor of Journal
Rosenwasser et al.^9^	1989	CS	20	Vestibular schwannoma (*n* = 14) Meningioma in the cerebellopontine angle (*n* = 4) Cerebellar hemangioblastoma (*n* = 1) Cerebellar metastasis (*n* = 1)	5	4.1
Slavin et al.^11^	2003	CS	5	Cerebellar hemorrhage (*n* = 1) Subarachnoid hemorrhage of posterior circulation (*n* = 2) Infratentorial arteriovenous malformation (*n* = 1) Infratentorial arachnoid cyst (*n* = 1)	4	2.4
Moyse et al.^5^	2016	CR	1	Cerebellar infarct (*n* = 1)	N/A	3.0
Khan et al.^4^	2020	CR	1	Vestibular schwannoma (*n* = 1)	N/A	1.3

*CS* case series, *CR* case report, *NOS* New Castle Ottawa Scale

Four other studies contained animals in a preclinical experimental setting including a total of 26 pigs and eight dogs [2, 9, 10, 14]. Of these four studies, three studies investigated mass lesions in the epidural frontal/temporal and basal ganglia region by the injection of autologous blood [6, 9, 14]. In one study, a balloon was installed in the cerebellum and was insufflated to imitate a mass lesion in the posterior fossa. Depending on the volume of the mass, the supratentorial (right frontal) ICP was compared with the infratentorial (cerebellar) ICP (Table 2)[10].

**Table 2:** Study characteristics (animals)

References	Year	Type	(n)	Mass location	Lesion (cc)	Supratentorial ICP (mmHg)	Infratentorial ICP (mmHg)	Impact factor of Journal
Wolfa et al.^18^	1996	pig	10	Right epidural frontal	1 2 3 4 5 6	7.2 10.3 15.8 24.8 29.9 41.6	6.3 7.4 8.8 9.9 10.4 12.3	4.0
Wolfa et al.^17^	1997	Pig	9	Right epidural temporal	0 3 6	10.5 59,8 147.9	9.7 39.2 57.5	4.0
Rieger et al.^8^	1999	Pig	7	Left cerebellar	0 Balloon infl	4.1 63.1	4.4 62.3	1.3
Qureshi et al.^7^	2002	Dog	8	Right basal ganglia	5	42.1 $\pm$ 3.5	29.1 $\pm$ 4.5	7.6

*ICP* intracranial pressure

### Meta-analysis of supra- and infratentorial ICP measurement

**Fig. 2:**
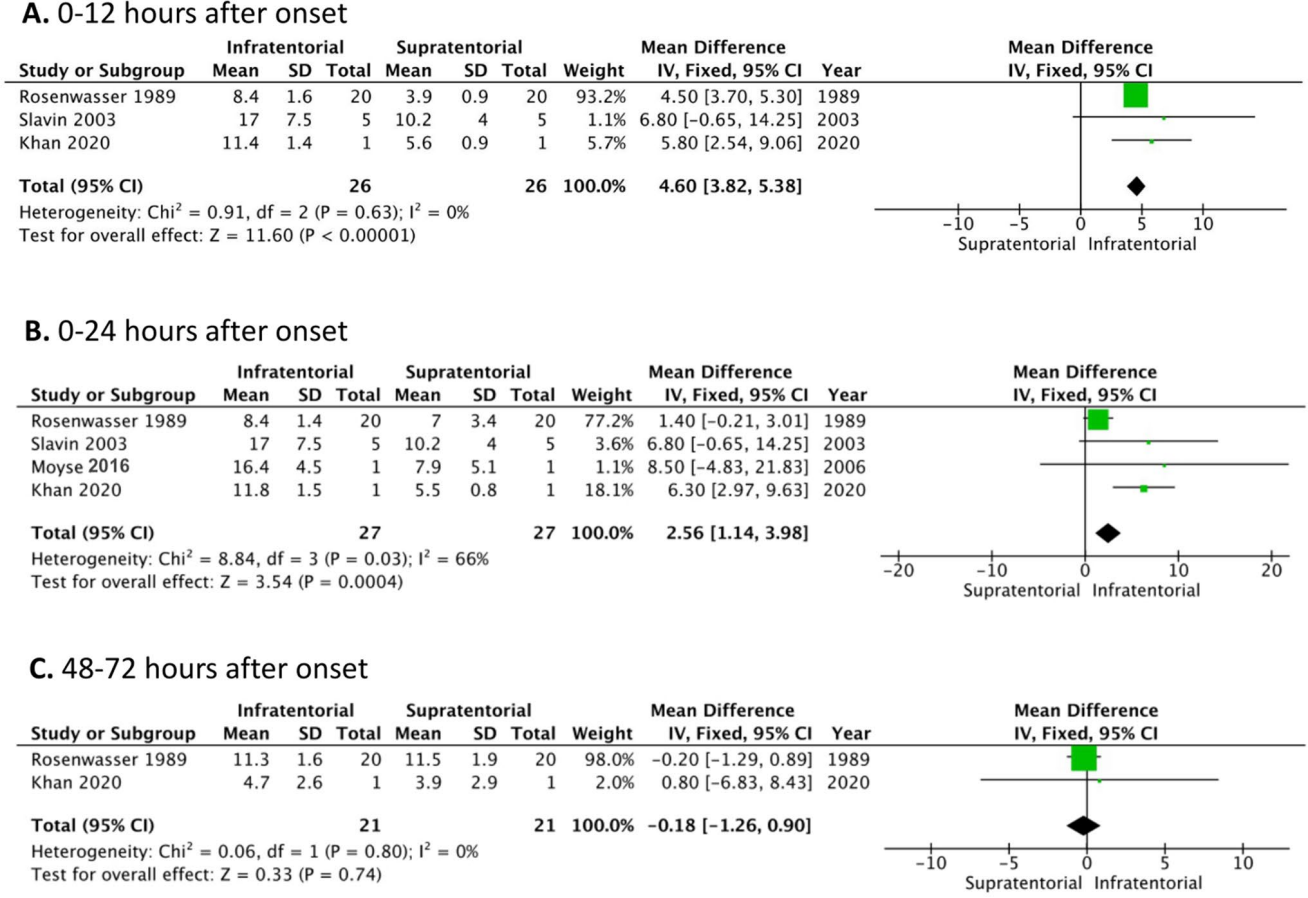
Meta-analysis comparing supra- and infratentorial intracranial pressure monitoring

Three of four studies reported simultaneous ICP measurement up to 12 h after onset (0–12 h). Consequently, a total of 26 patients were included in this analysis [7, 11, 12]. The pooled data demonstrated that the mean value of infratentorial ICP measurement was significantly higher than the supratentorial ICP measurement (**∆**ICP = 4.6 mm Hg 95% CI 3.82–5.38, *p* < 0.05, *I*^2^ = 0%) in favour for superiority of infratentorial measurement (Fig. 2a).

The second comparison with ICP measurement up to 24 h (*0–24 h*) included all four human studies with a total number of 27 patients [7, 8, 11, 12]. The pooled data demonstrated that there was again a significantly different mean value between supra- and infratentorial ICP (**∆**ICP = 2.56 mm Hg 95% CI 1.14–3.98, *p* < 0.05, *I*^2^ = 66%) in favor for superiority of infratentorial measurement **(**Fig. 2b).

The third comparison with ICP measurement between *48 and 72 h* included two studies with a total number of 21 patients [7, 11]. The pooled data demonstrated a non-statistically significant mean difference of 0.18 mmHg (95% CI − 1.26 to 0.9, *p* > 0.05, *I*^2^ = 0%) between supra- and infratentorial ICP measurement (Fig. 2c).

### Systematic review of supra- and infratentorial ICP measurement in an experimental setting

The supratentorial ICP correlated well with the amount of the mass effect on epidural supratentorial compartment [2, 9, 14]. Increasing value of infratentorial ICP was shown as well; however, there was a significant mismatch between supra- and infratentorial ICP (range 0.9–90.4 mmHg) [2, 14]. Similar result could be shown in case of intracerebral mass effect with a significant difference between the mean value of supra- and infratentorial ICP (42.1 $\pm$ 3.5 mmHg vs 29.1 $\pm$ 4.5 mmHg; *p* = 0.0009) [9]. In contrast, one study showed no difference between supra- and infratentorial ICP measurement in case of mass lesion in the cerebellar compartment (*p* > 0.05) [10].

## Discussion

The simultaneous supra- and infratentorial ICP measurement showed significant different values between those two compartments in human and in experimental models. The difference of those compartments persisted significantly in the first 48 h postoperatively, whereas after 48–72 h, both values equilibrated without any significant difference.

This manuscript addresses a potentially relevant transtentorial gradient with clinical indication for additional intervention in the posterior fossa compartment. The outstanding question is to select right patient collective who might benefit from monitoring of posterior fossa compartment. According to the Monro-Kellie doctrine composed of brain, liquor, and blood, the intracranial space is limited and a mass lesion results in the reduction of liquor and blood component until the herniation of brain occurs [16, 17]. For the simplicity of this model, it is assumed that the pressure measured in a single location reflects the pressure throughout the brain. However, several manuscript as well as our review show that there is a significant difference between supra- and infratentorial ICP in humans depending on the location of the lesion, volume of the lesion, and time after onset of the lesion [7, 8, 11, 12]. Similar results were shown in experimental studies including animals except for one study reporting non-significance of supra- and infratentorial ICP measurements [2, 9, 10, 14]. Not only between supra- and infratentorial compartment but also the more remotely ICH was measured, the more significant ICP difference occurred. For example, in case of mass lesion in the frontal lobe in an experimental setting, the ICP value decreased as follows: frontal lobe > temporal lobe > midbrain > cerebellum [2]. Accordingly, the meaning of infratentorial pressure monitoring appears to be both important and relevant in the clinical management of posterior fossa lesions, the larger the mass lesion the more important the investigation of posterior fossa ICP monitoring. One should be alert that a normal supratentorial ICP could mistakenly mask the pressure-related secondary injury in the posterior fossa region. Still, a supratentorial ICP monitoring is necessary due to its risk of development of hydrocephalus by obstruction of fourth ventricle. At the end, the composition of those pathophysiological mechanisms is the determining factor for the final outcome of patients.

Further, the type of mass lesion and the preoperative neurological status might be crucial for the indication of ICP monitoring in the posterior fossa region. Cerebellar hemorrhage or infarction is lesion with increased risk for postoperative edema development or rebleeding compared to elective tumor surgery, which might be appropriate for an infratentorial ICP monitoring. In addition, good preoperative neurological status is a reliable parameter to compare the postoperative result independent from an ICP monitoring, whereas worse preoperative neurological status might end up in difficulty to evaluate the actual status of patients. We think that those patients might benefit from an ICP monitoring in the posterior fossa region.

Even focusing on the supratentorial compartments alone, there are contrary reports in the literature concerning interhemispheric gradient. Several studies observed significantly higher ICP values on the ipsilateral side of the mass lesions compared to the contralateral side concluding the existence of interhemispheric ICP gradients. With time, the gradient disappeared and both values equilibrated [2, 18, 19]. In contrast, Yano et al. observed bifrontal ICPs in patients with traumatic brain injury showing no significant difference in the concurrent comparative ICPs concluding that the supratentorial space be assumed as one compartment regardless of different types of intracranial lesions [20]. As a limitation of this study, the observed range of ICPs was between − 3 and 130 mmHg. Based on the results, it again strengthens the hypothesis that two factors are relevant concerning the gradient between different compartments: the size of mass lesion and time after onset of the lesion.

After a period of time, the intracranial pressure in different compartment begins to equilibrate, and according to our meta-analysis, the threshold was between 24 and 48 h after onset. In other words, we have a blind window of 48 h without showing the actual pressure of posterior fossa compartment if only a supratentorial ICP measurement is performed. The aspects unique to the posterior fossa include the limited volume, a close relationship to the brain stem, and a high probability of development of occlusive hydrocephalus. Giving the truth of insufficient supratentorial ICP measurement for 48 h, secondary brain injury in this region could lead to a fatal outcome. Therefore, a simultaneous monitoring of supra- and infratentorial ICP measurement is important at least for 48 h or more in patients with mass lesion in the posterior fossa. The time window might depend on the underlying pathology (cerebellar tumor, hemorrhage, infarction) and volume of the mass lesion, wherefore further studies need to be investigated to evaluate the necessary time window for simultaneous ICP measurement.

As reported, there are several studies reporting a difference in pressures between various compartments of the brain. However, there are no studies reporting the relationship between infratentorial ICP monitoring and the outcome of patients. This should be addressed in future studies to establish an evidence-based treatment strategy for posterior fossa pathologies to promote better ICP monitoring and outcome of our patients.

## Limitations

This study contains human and animal studies, which are mostly based on case series or case reports. Accordingly, the quality of included studies is limited. However, the main conclusions of those studies are uniform which show indirectly the relevance of further prospective studies to evaluate this issue more in detail. Secondly, the simultaneous ICP measurement was performed in different pathologies in the posterior fossa region. This is an important point, since the difference of supra- and infratentorial ICP measurement as well as the time window until the pressure equilibration might be more prominent if the pathology of posterior fossa region was more space-occupying.

## Conclusions

Supratentorial ICP measurement is not a reliable tool to reflect the pressure in the posterior fossa. Therefore an infratentorial ICP monitoring might be mandatory to reflect the real pressure in the posterior fossa region to prevent further secondary brain injury. Simultaneous supra- and infratentorial ICP measurement should be performed at least for 48 h or more until the pressure begins to equilibrate.

## Data Availability

The datasets used and/or analyzed during the current study are available from the corresponding author on reasonable request.
